# C-Reactive Protein to Albumin Ratio as A Novel Inflammatory-Based Marker for 30-Day Mortality in Patients Undergoing Transcatheter Aortic Valve Replacement

**DOI:** 10.21470/1678-9741-2020-0482

**Published:** 2022

**Authors:** Fahrettin Katkat, Muhsin Kalyoncuoglu, Sevgi Ozcan, Sevil Tugrul, Hanife Abanus, Orhan Ince, Mehmet Balli, Irfan Sahin, Ertugrul Okuyan

**Affiliations:** 1 Cardiology Department, Bagcilar Training and Research Hospital, University of Health Sciences, Istanbul, Turkey.; 2 Cardiology Department, Haseki Training and Research Hospital, University of Health Sciences, Istanbul, Turkey.; 3 Cardiology Department, Mersin Training and Research Hospital, University of Health Sciences, Istanbul, Turkey.

**Keywords:** Transcatheter Aortic Valve Replacement, Serum Albumin, C-Reactive Protein, Mortality, Aortic Stenosis

## Abstract

**Objective:**

We aimed to investigate whether C-reactive protein to albumin ratio (CAR) predicts the early and late mortality in patients undergoing transcatheter aortic valve replacement (TAVR).

**Methods:**

This study was retrospectively designed and includes 170 TAVR patients with a mean age of 78.4±7.1 years. Patients were divided into 2 groups as those who died and those who survived, taking into account the follow-up period. Complete blood count, serum CRP and serum albumin were obtained on admission. The CAR value of all patients was calculated and the relationship of CAR with early (≤30 days) and late mortality (>30 days) was evaluated.

**Results:**

The median follow-up period was 19 [7-31] months (maximum 66 months). Early mortality was observed in 20 (11.8%) patients, whereas late mortality was observed in 39 (22.9%) patients, most of them male (61.1%, *P*=0.04). Non-survivors had greater CAR value, higher baseline serum CRP level and lower baseline albumin level than survivors (*P*<0.01, for all parameters). According to multivariate analysis models, CAR (HR: 1.020, *P*<0.01) and TVAR score (HR: 1.294, *P*<0.01) were found to be independent predictors of early mortality while CRP and albumin were not. The area under the curve (AUC) for CAR was 0.73 with a *P* <0.01. A CAR >15.6 predicted the early mortality with 80% sensitivity and 57% specificity.

**Conclusion:**

The novel inflammatory marker CAR can be used as a reliable marker in predicting 30-day mortality in patients undergoing TAVR.

**Table:** Abbreviations, acronyms & symbols

AS	= Aortic stenosis
AUC	= Area under the curve
AVR	= Aortic valve replacement
CAR	= C-reactive protein-to albumin ratio
CI	= Confidence interval
CRP	= C-reactive protein
CVA	= Cerebrovascular accident
HR	= Hazard ratio
LVEF	= Left ventricular ejection fraction
NYHA	= New York Heart Association
ROC	= Receiver operating characteristic
TAVR	= Transcatheter aortic valve replacement
VARC-2	= Valvular Academic Research Consortium-2

## INTRODUCTION

Transcatheter aortic valve replacement (TAVR) is well-established treatment option for patients with symptomatic severe aortic stenosis (AS) who are not candidates for surgery or who are considered to be at high surgical risk^[1]^. Although various aetiological risk factors for degenerative AS have been identified, it has been described as an inflammatory-based disease that shares common pathophysiology with atherosclerosis, including calcification, accumulation of lipoproteins and chronic inflammation^[2]^. Because AS is the result of a dynamic inflammatory process, not just ageing, it has been suggested that inflammatory biomarkers can predict outcomes in patients receiving TAVR^[3]^. It has been reported that persistently elevated levels of circulating plasma inflammatory proteins following TAVR are associated with increased cardiovascular and all-cause mortality^[4]^.

C-reactive protein (CRP) is a nonspecific acute-phase reactant that is classically related to inflammation and is thought to play a critical role in both progression and severity of degenerative AS^[2,5]^. Although elevated CRP level was found to be associated with increased risk of subsequent aortic valve replacement^[6]^, there are conflicting data about the prognostic importance of high CRP level in patients who have undergone TAVR^[2,5,7]^. Serum albumin is a negative acute-phase reactant and, as a marker of malnutrition, is part of the frailty criteria^[8]^. There is evidence that serum albumin is associated with an increased risk of cardiovascular disease mortality in various clinical conditions and in patients who undergo TAVR^[9]^. Recently, a newly defined inflammatory marker, the CRP to albumin ratio (CAR), has been found to be a more valuable predictive biomarker than either CRP or albumin alone due to its incorporation of two measures (increased CRP and decreased albumin) to predict inflammatory status and prognosis in various clinical settings^[10,11]^. It has also been proposed that CAR is strongly associated with increased mortality rates in patients undergoing surgical aortic valve replacement (AVR)^[12]^.

To our knowledge, there are no data available on the prognostic accuracy of CAR in patients who underwent TAVR. Therefore, in this study, we sought to investigate the predictive role of CAR in determining early and late mortality in patients who underwent TAVR.

## METHODS

One hundred eighty-four consecutive patients who underwent TAVR were retrospectively analysed. Data collected as a part of routine clinical practice at Bagcilar Training and Research Hospital between January 2015 and December 2019 and compiled in a database were reviewed. Data from each patient, including clinical assessment, electrocardiogram, chest radiography, echocardiogram, multislice computed tomography of the aorta and branches, cine coronary angiography and laboratory tests, were obtained from a computerised system, patient file records and/or patients’ follow-up visits.

Severe AS is defined as by an aortic valve area of <1.0 cm^2^ and/or <0.6 cm^2^/m^2^, and/or a mean transaortic pressure gradient of >40 mmHg and/or a peak aortic jet velocity >4.0 m/s. Exclusion criteria of the current study were simultaneous percutaneous coronary intervention (n=2), life expectancy shorter than 1 year (n=3), severe mental impairment (n=2) and lack of data (n=7). Following the exclusion criteria, the remaining 170 patients with a life expectancy of at least 1 year who were considered to be at high risk based on clinical assessments by a multidisciplinary heart team constituted the study population^[1]^. The heart team used a guideline based on a risk model developed by the EuroSCORE II (ES II) to estimate the risk of death after the index TAVR procedure. EuroSCORE II and ACC/STS TAVR scores were calculated using online tools (www.euroscore.org and https://tools.acc.org/tavrrisk)

The default access for TAVR was transfemoral; alternative routes (transapical, carotid and subclavian) were used in only 3 patients. The valve choice was at the discretion of the heart team. The procedure was performed in the cardiac catheterisation laboratory under general anaesthesia or sedoanalgesia with transoesophageal echocardiography guidance. All patients received aspirin (81 mg) and clopidogrel (≥300 mg) before the procedure and heparin during the procedure; patients continued to take aspirin indefinitely and clopidogrel for a minimum of 1 month. After the index procedure, all patients were followed up for 30 days, 6 and 12 months and yearly thereafter. Since our study was retrospectively designed, written informed consent from participants could not be obtained, but our study protocol was approved by the Ethics Committee of our institution.

Ethical approval for this study was obtained from Bagcilar Training and Reseach Hospital Ethics Committee under number: 2020.02.1.10.028.

### Laboratory Measurements

Routine complete blood cell count and blood evaluations for determining glucose, creatinine, albumin and CRP levels were performed using the admission blood samples. Serum albumin and CRP levels were measured using a Roche Diagnostics Cobas 8000 c502 analyser (Roche Holding AG, Basel, Switzerland). CAR was calculated as the ratio of serum CRP level (mg/L) to serum albumin level (mg/L), multiplied by 100 to facilitate interpretation, as done in previous studies^[12]^. The estimated glomerular filtration rate (eGFR) was calculated using the Modification of Diet in Renal Disease formula.

### Study Endpoint

Outcomes (overall mortality, cardiovascular mortality, pacemaker requirement, overall bleeding, major bleeding, cerebrovascular accident, major vascular complications and overall vascular complications) were adjudicated according to Valvular Academic Research Consortium-2 (VARC-2) criteria^[13]^. The primary endpoint of the study was all-cause mortality within 30 days (defined as early mortality) and at more than 30 days (defined as late mortality) for the study cohort. The follow-up rate was 100% for all patients after the index operation. All events were independently reviewed by an independent group of clinicians who were blinded to patients’ baseline CRP values.

### Statistical Analysis

The continuous variables were given as mean ± standard deviation if the data were normally distributed and median (interquartile range) if the data did not have a normal distribution. The categorical variables were presented as percentages. The Chi-square (χ^2^) test was used to compare the categorical variables between the groups. The Kolmogorov-Smirnov test was used to assess whether the variables were normally distributed. Student’s *t*-test or Mann-Whitney U test was used to compare the continuous variables between groups according to whether data were normally distributed. To identify predictors of early and late mortality, univariate and multivariate Cox proportional hazards regression analyses were performed. For all regression analyses, only variables with a *P*<0.1 in univariate analysis were incorporated into the multivariate model, with results reported as the hazard ratio (HR) and 95% confidence interval (CI). Variables already included in the ACC/STS TAVR score were not considered separately in multivariate analysis, independent of their significance in univariate analysis. Receiver operating characteristic (ROC) curve analysis was used to evaluate the sensitivity and specificity of the CAR and its optimal cut-off value (Youden index) for predicting early mortality. Kaplan-Meier survival curves were used to depict the early survival pattern of patients who were stratified into high- and low-risk groups using the cut-off CAR score ≥15.6 in our cohort. All statistical analyses were carried out using the Statistical Package for the Social Sciences version 24.0 (IBM Corp., Armonk, NY, USA).

## RESULTS

### Baseline Characteristics

The median follow-up period was 19 (7-31) months, with a maximum of 66 months. The mean age was 78.4±7.1 years and 85 (50%) patients were male. Admission diagnosis was heart failure in 122 (71.5%), angina or angina equivalent symptoms in 237 (22.0%) and presyncope or syncope in 11 (6.5%), and none of the patients suffered sudden cardiac death. Mean aortic valve area was 0.72±0.15 cm^2^ and the mean transvalvular gradient was 48.2±9.0 mmHg. Seventy-four (43.5%) patients had severe symptoms, defined as New York Heart Association (NYHA) class III/IV. Median ES II was 5.0 (3.4-9.9), and median TAVR score was 3.9 (2.6-5.3). Compared to surviving patients, patients who died had higher CAR values (28 [12-68] *vs*. 11 [7-28], *P*<0.01), higher basal serum CRP levels (10 [4.7-24] *vs*. 4.6 [3-11.6], *P*<0.01) and lower basal albumin levels (3.7±0.32 *vs*. 4±0.32, *P*<0.01). Detailed demographic, clinical, laboratory and echocardiographic characteristics of the population are summarised in Table 1.

**Table 1:** Baseline demographic, clinical, laboratory and echocardiographic parameters of study population.

Variables	All population	Alive	Dead	*P*-value
		n=111 (65.3%)	n=59 (34.7%)	
Male gender, n (%)	85 (50)	49 (44.1)	36 (61.1)	0.04
Age, years	78.4±7.1	77.9±7.1	79.5±7.0	0.17
BMI, kg/m^2^	26.8±4.2	26.9±4.4	26.5±3.6	0.6
Hypertension, n (%)	106 (62.4)	74 (66.7)	32(54.2)	0.11
Diabetes mellitus, n (%)	80 (47.1)	50 (45)	30 (50.8)	0.47
Hyperlipidaemia, n (%)	94 (55.3)	65(58.6)	29 (49.2)	0.24
Smoking, n (%)	86 (50.6)	52 (47.3)	34 (57.6)	0.2
Vascular disease history, n (%)	95 (55.9)	58 (52.3)	37 (62.7)	0.19
CVA history, n (%)	17 (10)	4 (3.6)	13 (22)	<0.01
COPD, n (%)	75 (44.1)	46 (41.4)	29 (49.2)	0.34
NYHA class III-IV, n (%)	74 (43.5)	36 (32.4)	38 (64.4)	<0.01
Atrial fibrillation, n (%)	31(18.2)	18 (16.2)	13 (22)	0.35
Presence of BBB, n (%)	26 (15.3)	19(17.1)	7(11.9)	0.37
Aortic valve area, cm^2^	0.72±0.15	0.71±0.15	0.73±0.15	0.40
Mean aortic valve gradient, mmHg	48.2±9	47.8±8.6	48.9±9.9	0.45
Left ventricular ejection fraction, %	51.7±9.7	53.1±8.8	49.1±10.8	0.01
sPAP, mmHg, [IQR]	36 [32-45]	36 [30-45]	38 [35-45]	0.15
TAVR score, %, [IQR]	3.9[2.6-5.3]	3.0 [2.3-4.3]	5.3 [4.2-7.7]	<0.01
EuroSCORE II	4.9 [3.4-9.1]	3.9 [2.6-6.7]	7.4 [4.4-11.9]	<0.01
FBG, mg/dL	1201[104-150]	123 [105-152]	119 [103-146]	0.75
eGFR, mL/min	68 [49-80]	71 [52-80]	56 [44-77]	<0.01
Haematocrit, %	36.1±5.5	36.4±5.5	35.8±5.5	0.48
WBC, 10^3^/µL	7.4±2.1	7.4±1.9	7.4±2.4	0.84
Platelet, 10^3^/µL	230.5±80.1	236.2±83.9	219.8±71.9	0.21
CRP	5.8 [3.2-15.8]	4.6 [3-11.6]	10 [4.7-24]	<0.01
Albumin, g/dL	3.9±0.34	4±0.32	3.7±0.32	<0.01
CARX100	14 [8-39]	11 [7-28]	28 [12-68]	<0.01

BBB=bundle branch block; BMI=body mass index; CAR=C-reactive protein to albumin ratio; COPD=chronic obstructive pulmonary disease; CRP=C-reactive protein; CVA=cerebrovascular accident; eGFR=estimated glomerular filtration rate; EuroSCORE II=European System for Cardiac Operative Risk Evaluation II; FBG=fasting blood glucose; IQR=interquartile range; NYHA=New York Heart Association; sPAP=pulmonary artery systolic pressure; TAVR=transcatheter aortic valve replacement; WBC=white blood count

The procedure was performed using conscious sedation in 146 (85.9%) patients and general anaesthesia in 24 (14.1%) patients. Seventy-one (41.8%) patients received the PORTICO (St. Jude Medical; Minneapolis, MN, USA), 56 (32.9%) received the Edwards-SAPIEN XT (Edwards Lifesciences), 20 (11.8%) received the EVOLUTE-R (Medtronic Inc.) and 23 (13.6%) patients received other valve types, including Direct Flow and Acurate valves. Among the major VARC-2-defined procedure-related complications, 19 (11%) patients needed new pacemaker insertion, 25 (14.7%) had any VARC-2-defined major vascular injury, 41 (24.1%) experienced major bleeding and 6 (3.5%) suffered cerebrovascular accident. In addition, acute kidney injury developed in 41 patients and myocardial infarction occurred in 2 patients. None of the patients needed surgical intervention peri-TAVI or post-TAVI. Periprocedural characteristics of the study population are summarised in Table 2.

**Table 2:** Procedural and postprocedural parameters of study population during the follow-up period.

Variables	All population	Alive	Dead	*P*-value
		n=111 (65.3%)	n=59 (34.7%)	
Type of valve, n (%)				0.1
EVOLUT R	20 (11.8)	11 (9.9)	9 (15.3)	
SAPIEN XT	56 (32.9)	31 (27.9)	25 (42.4)	
PORTICO	71 (41.8)	50 (45)	21 (35.6)	
ACURATE	12 (7.1)	11 (9.9)	1 (1.7)	
DIRECT FLOW	11 (6.5)	8 (7.2)	3 (5.1)	
Predilatation, n (%)	98 (57.6)	65 (58.6)	33 (55.9)	0.74
Post dilatation, n (%)	24 (14.1)	16 (14.4)	8 (13.6)	0.88
Implantation depth, mm	5.26±0.75	5.29±0.77	5.22±0.73	0.59
Paravalvular leakage (>2+), n (%)	13 (7.6)	7 (6.3)	6 (10.2)	0.37
Major vascular complications, n (%)	25 (14.7)	16 (14.4)	9 (15.3)	0.88
Bleeding complications, n (%)	41 (24.1)	25 (22.5)	16 (27.1)	0.51
Pericardial tamponade, n (%)	5 (2.9)	0 (0)	5 (8.5)	<0.01
Acute kidney injury, n (%)	41 (24.1)	16 (14.4)	25 (42.4)	<0.01
Permanent pacemaker, n (%)	19 (11.1)	14 (12.6)	5 (8.5)	0.42
Rehospitalisation, n (%) (cardiovascular cause)	35 (20.5)	17 (15.3)	18 (30.5)	0.02
Sepsis with worsening of heart function, n (%)	0	0	0	0
Poor positioning of the prosthesis/thrombosis, n (%)	5 (2.9)	2 (1.8)	3 (5.1)	0.23
Postprocedural IS or TIA, n (%)	6 (3.5)	5 (4.5)	1(1.7)	0.35
Myocardial infarction, n (%)	2 (1.2)	2 (1.8)	0 (0)	0.3
Infective endocarditis, n (%)	0	0	0	0

IS=ischemic stroke; TIA=transient ischemic attack

### Parameters Associated with Mortality

During the follow-up period, 59 patients died. Twenty (11.8%) deaths occurred within 30 days, and 39 (22.9%) in a later period (>30 days). Death was more common in male patients (*P*=0.04). Patients who died had higher frequencies of cerebrovascular accident (CVA, *P*<0.01) and NYHA class III-IV (*P*<0.01) than survivors. As revealed in echocardiographic examinations, left ventricular ejection fraction (LVEF) was lower in patients who died (*P*<0.01), whereas mean aortic valve area, mean aortic valve gradient and pulmonary artery systolic pressure were not different between the groups (*P*>0.05 for all). Compared to those who survived, those who died had lower eGFR and serum albumin (*P*<0.01 and *P*<0.01, respectively) and higher initial serum CRP levels and CAR values *P*<0.01 and *P*<0.01, respectively). Furthermore, ES II and TAVR score were significantly higher in patients who died than in survivors (*P*<0.01 and *P*<0.001, respectively). Additionally, pericardial tamponade, acute kidney injury and rehospitalisation due to cardiovascular conditions were more frequent in patients who died (*P*<0.01, *P*<0.01 and *P*=0.02, respectively). Detailed clinical, laboratory and procedural features of both groups are summarised in Tables 1 and 2.

### Independent Predictors of Early-Term Mortality

**Fig. 1:**
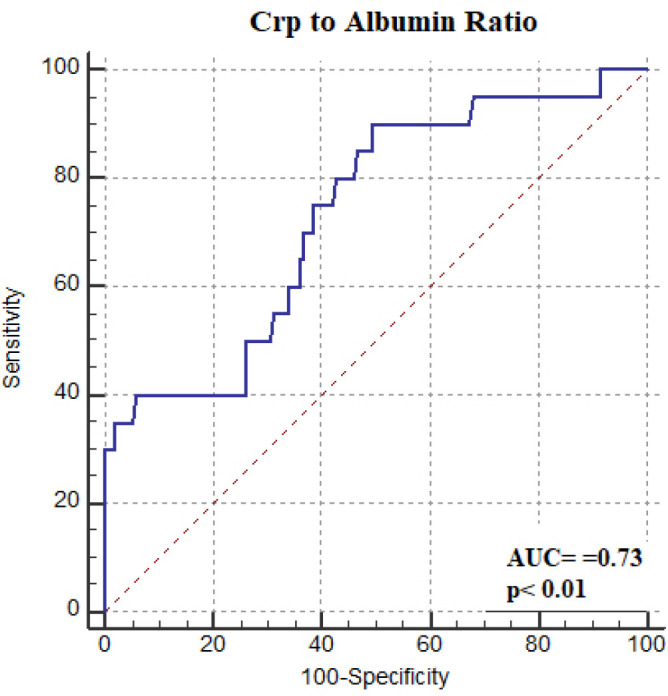
ROC curve of CAR score for detecting early mortality.

**Fig. 2:**
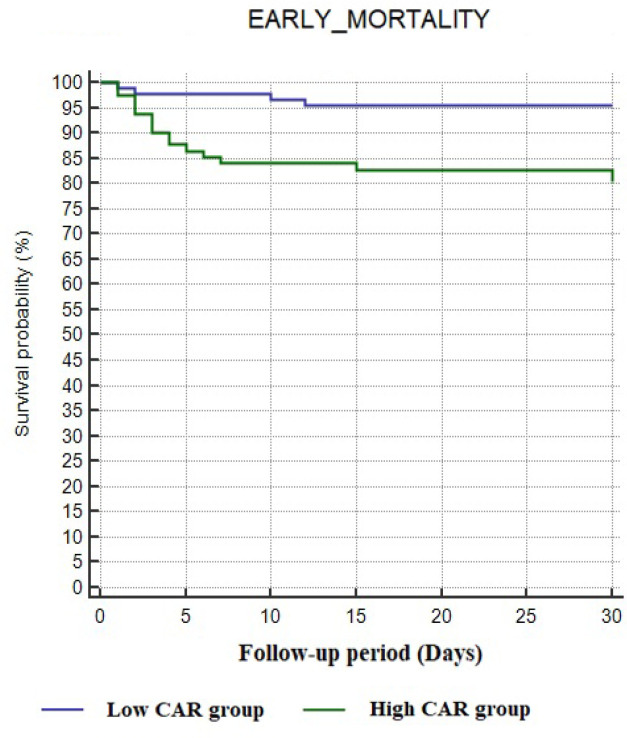
Kaplan-Meier plots of survival curves of patients with low (blue line) and high (green line) CAR score categories.a

Decreased LVEF, higher TAVR score, higher serum CRP level, hypoalbuminemia, higher CAR value and postprocedural acute kidney injury were associated with early mortality in univariate Cox regression analysis (*P*<0.05). To determine the independent predictors of mortality using variables that exhibited statistically significance in the univariate analysis, we performed two multivariate Cox regression analyses, as we believed that CRP and albumin alone or together would negatively affect the statistical significance of CAR in predicting early mortality. In addition, parameters such as age and NYHA class in the TVAR scoring system were not included in the regression analysis. Furthermore, ES II is mainly used in clinical practice to estimate surgical risk prior to surgical AVR and is not a TAVR-specific scoring system to stratify patients undergoing TAVR^[14]^. Thus, ES II score was not included in the multivariate regression model. On the other hand, the STS/ACC TAVR risk score, which was recently developed to predict the risk of in-hospital mortality in patients undergoing TAVR, was included in the regression analysis model. Additionally, since tamponade was observed in only a small number of patients (n=5), we did not conduct a statistical analysis for in-hospital mortality. CAR was a statistically significant predictor of mortality in model 1 (HR: 1.020, *P*<0.01), while CRP and albumin levels alone were not found to be independent predictors of early mortality in model 2 (*P*=0.20 and *P*=0.12, respectively). In addition, in both Cox regression analysis models, the TAVR score was found to be an independent predictor for early mortality (HR: 1.294, *P*<0.01 and HR: 1.331, *P*<0.01, respectively; Table 3). To test the predictive performance of CAR, we performed ROC curve analysis. The area under the curve for 30-day mortality was 0.73 (95% CI: 0.62-0.85, *P*<0.01) with a cut-off value of 15.6 (80% sensitivity, 57% specificity; Figure 1). The Kaplan-Meier curves in Figure 2 represent mortality in patients divided into low-risk (CAR <15.6) and high-risk (CAR ≥15.6) groups during the 30-day follow-up.

**Table 3:** Two different univariable and multivariable Cox proportional hazards regression analysis models for determining predictors of 30-day and >30-day mortality.

	Univariable HR (95% CI)	*P*-value	Model 1	*P*-value	Model 2	*P*-value
			Multivariable		Multivariable 2	
Early mortality			HR (95% CI)		HR (95% CI)	
Acute kidney injury	2.624 (1.087-6.333)	0.03	1.171 (0.401-3.423)	0.77	0.974 (0.317-2.991)	0.97
LVEF	0.964 (0.928-1.000)	0.05	0.982 (0.946-1.021)	0.36	0.985 (0.949-1.023)	0.44
TAVR score	1.407 (1.229-1.611)	<0.01	1.294 (1.092-1.533)	<0.01	1.331 (1.115-1.589)	<0.01
CAR	1.026 (1.013-1.039)	<0.01	1.020 (1.006-1.034)	< 0.01	-	-
CRP	1.055 (1.018-1.093)	<0.01	-	-	1.030 (0.985-1.078)	0.20
Albumin	0.827 (0.727-0.940)	<0.01	-	-	0.889 (0.767-1.031)	0.12
Late mortality						
CVA history	4.332 (2.048-9.166)	<0.01	4.985 (2.121-11.718)	<0.01	4.738 (2.004-11.200)	<0.01
TAVR score	1.410 (1.258-1.581)	<0.01	1.249 (1.080-1.444)	<0.01	1.241 (1.073-1.435)	<0.01
LVEF	0.952 (0.925-0.980)	<0.01	0.954 (0.924-0.984)	<0.01	0.953 (0.924-0.984)	<0.01
Acute kidney injury	3.868 (1.994-7.503)	<0.01	2.240 (1.060-4.733)	0.04	2.189 (1.024-4.680)	0.04
Rehospitalisation	2.058 (1.068-3.966)	0.03	1.636 (0.805-3.322)	0.17	1.565 (0.747-3.277)	0.24
CAR	1.019 (1.007-1.033)	<0.01	1.008 (0.993-1.023)	0.29	-	-
CRP	1.042 (1.009-1.077)	0.01	-	-	1.021 (0.979-1.066)	0.33
Albumin	0.867 (0.782-0.960)	<0.01	-	-	0.959 (0.853-1.079)	0.49

CAR=C-reactive protein to albumin ratio; CRP=C-reactive protein; CVA=cerebrovascular accident; LVEF=left ventricular ejection fraction; TAVR=transcatheter aortic valve replacement

### Independent Predictors of Late Mortality

Univariate analysis revealed that decreased LVEF, higher TAVR score, higher serum CRP level, hypoalbuminemia, higher CAR value and postprocedural acute kidney injury were also associated with late mortality. Moreover, in addition to these parameters, late mortality was associated with CVA history and cardiovascular hospitalisation. Both multivariate regression analysis models (1 and 2) showed that CVA history (HR: 4.985, *P*<0.01 and HR: 4.738, *P*<0.01, respectively), higher TAVR score (HR: 1.249, *P*<0.01 and HR: 1.241, *P*<0.01, respectively), lower LVEF (HR: 0.954, *P*<0.01 and HR: 0.953, *P*<0.01, respectively) and the development of postprocedural acute kidney injury (HR: 2.240, *P*<0.01 and HR: 2.189, *P*<0.01, respectively) were independent predictors for late mortality (Table 3).

## DISCUSSION

The main findings of the present study are as follows: (i) elevated baseline serum CRP and low serum albumin levels are associated with early and late mortality, but these are not predictors of short- or late-term mortality; (ii) although there is an association between short- and long-term mortality and CAR, CAR was found to be a predictor of only short-term mortality; and (iii) patients with a CAR score >15.6 points are at high risk of adverse early-term cardiovascular outcomes.

AS is a degenerative valvular disease characterised by an inflammatory response and an active process of atherosclerosis, calcification and ossification^[15]^. TAVR is increasingly being used as an alternative to surgical AVR in the treatment of symptomatic patients with severe AS at high surgical risk or who are inoperable^[1]^. It has been suggested that AS is associated with a dynamic inflammatory process, not simply a result of ageing alone, and that increased levels of inflammatory biomarker before and after TAVR may predict outcomes in patients treated with TAVR^[3,4,16]^.

CRP is a marker of systemic inflammation and has been proposed to be increased in patients with degenerative AS, similar to the increase found in patients with atherosclerosis^[17]^. There are also studies suggesting the prognostic importance of CRP in various cardiovascular diseases^[14,18]^ and outcomes after cardiovascular interventions such as percutaneous coronary intervention and coronary artery bypass grafting^[19,20]^. There have also been several previous studies investigating the prognostic value of CRP in patients undergoing TAVR^[3,5,16,21]^. Similar to the results of our study, Ruparelia et al.^[5]^ found that baseline CRP levels did not predict 30-day mortality in patients who underwent TAVR^[5]^. In contrast to our study, Hioki et al.^[16]^ found that high CRP on admission was significantly associated with an increased risk of all-cause mortality in the first 3 months after TAVR. However, consistent with the current study, they also demonstrated that CRP is not a predictor of long-term mortality (>6 months). Additionally, in line with our study, another study also demonstrated that baseline CRP value did not predict one-year mortality^[22]^. Overall, the results of the previous studies on the prognostic importance of CRP in predicting short- and long-term mortality in TAVR patients are conflicting^[3,5,7,16,22]^. The impact of CRP on mortality may be linked to the burden of co-morbidities (coronary artery disease, diabetes, atrial fibrillation, etc.) with chronic inflammation. Moreover, several previous studies suggested that patients with high CRP levels have more severe conditions and comorbidities such as diabetes, atrial fibrillation and vascular disease^[3,23]^. In the present study, when the patient population was evaluated in terms of comorbidities associated with chronic inflammation, no statistical difference was observed between the groups. This may be a reason why CRP does not predict short- and long-term mortality.

Serum albumin, as a negative acute-phase reactant, is a marker of systemic inflammation and has previously been shown to be associated with an increased risk of cardiovascular disease mortality in various patient subgroups^[9,11,24]^. Moreover, as a marker of malnutrition, serum albumin is part of the frailty criteria^[9]^. In contrast to the current study, several previous studies proposed that preprocedural serum albumin level was independently associated with mortality in patients who underwent TAVR. In addition, we know that serum albumin levels are influenced by vascular injury, renal injury, and levels of various cytokines. It also has an important role in controlling serum electrolyte levels and has antioxidant effects. Additionally, it can interact with free fatty acids and steroid hormones^[25]^. Due to these various functions, beyond its being a marker associated with mortality, it is very difficult to determine the direct relationship between serum albumin level and mortality and/or the exact mechanism of this relationship.

First described by Fairclough et al.^[10]^, CAR has been reported to have a favourable prognostic value in elderly patients with acute exacerbations of chronic disease and is a better predictor of long-term mortality in intensive care unit patients than CRP levels alone. It has also been suggested that it is more valuable than either serum CRP or albumin level alone in predicting the inflammatory status and prognosis in various clinical conditions^[10,11]^. Adverse clinical events are more prominently seen in the chronic inflammatory state in various clinical conditions^[10,12]^. Literature data reveal that increased inflammation is harmful for the heart endothelium and valve tissue, as seen in coronary atherosclerosis. Therefore, cardiac valvular degeneration can be seen more distinctly, and its progression and prognosis can be worse. A higher inflammatory state is also associated with an increased risk of mortality even if the valvular disease is treated. Recently, in a study by Kahraman et al.^[12]^, an elevated baseline CAR level was found to be associated with increased mortality rates in patients with isolated severe degenerative AS after surgical AVR. The authors also observed a higher rate of rehospitalisation due to cardiovascular causes in patients with increased CAR. To the best of our knowledge, this is the first study to verify the clinical importance of a high CAR on admission for early mortality after TAVR. Since acute-phase reactants do not exhibit similar responses for each inflammatory status, inflammation-based prognostic scores were found to provide more stable regulation. CAR contains both CRP and albumin parameters and reflects not only the proinflammatory state, but also nutritional status. As a prognostic score, this combination also provides stability between fluctuating CRP and albumin levels in diseases in which inflammation plays an important role. As a result, the combination of albumin and CRP in a single index may be more valuable than either measure alone and provide both inflammatory and nutritional information.

### Limitations

Our study has some limitations that should be noted. First, this study employed a retrospective design, had a relatively small sample size and relied on the experience at a single centre. Since our patient number was relatively small, this study may be underpowered to detect the predictive value of CAR on long-term mortality. Second, we measured only baseline CRP and albumin levels on admission, and changes that would be observed by serial measurements may have an additional predictive value. Third, we did not observe an association between the baseline CAR value and the major adverse events, such as pacemaker requirement, major vascular complications and cerebrovascular accident. Finally, the unknown aetiology of mortality was another important limitation in the study group due to the retrospective analysis of follow-up visits.

## CONCLUSION

Our findings suggest an independent role of CAR in early mortality in patients with isolated severe degenerative AS after TAVR beyond conventional inflammatory markers. From a clinical standpoint, we think that preoperative CAR is an easy, inexpensive and promising predictive inflammatory parameter and may be a part of the cardiovascular examination to identify high-risk individuals for the TVAR procedure. CAR could assist clinicians in their decision-making and may advise individual patients of their risk due to its ability to predict early mortality in patients undergoing TAVR. However, to evaluate the predictive value of CAR, and especially its prognostic value in patients who underwent TAVR, large-scale and prospective studies are still required.

**Table:** Authors' roles & responsibilities

FK	Substantial contributions to the conception or design of the work; or the acquisition, analysis or interpretation of data for the work; drafting the work or revising it critically for important intellectual content; final approval of the version to be published
MK	Substantial contributions to the conception or design of the work; or the acquisition, analysis or interpretation of data for the work; drafting the work or revising it critically for important intellectual content; final approval of the version to be published
SO	Substantial contributions to the conception or design of the work; or the acquisition, analysis or interpretation of data for the work; drafting the work or revising it critically for important intellectual content; final approval of the version to be published
ST	Substantial contributions to the conception or design of the work; or the acquisition, analysis or interpretation of data for the work; drafting the work or revising it critically for important intellectual content; final approval of the version to be published
HA	Substantial contributions to the conception or design of the work; or the acquisition, analysis or interpretation of data for the work; drafting the work or revising it critically for important intellectual content; final approval of the version to be published
OI	Substantial contributions to the conception or design of the work; or the acquisition, analysis or interpretation of data for the work; drafting the work or revising it critically for important intellectual content; final approval of the version to be published
MB	Drafting the work or revising it critically for important intellectual content; final approval of the version to be published
IS	Substantial contributions to the conception or design of the work; or the acquisition, analysis or interpretation of data for the work; drafting the work or revising it critically for important intellectual content; final approval of the version to be published
EO	Substantial contributions to the conception or design of the work; or the acquisition, analysis or interpretation of data for the work; drafting the work or revising it critically for important intellectual content; final approval of the version to be published
